# The Next Frontier in Aesthetics: 3D Bioprinting for Personalized Skin Regeneration

**DOI:** 10.1111/jocd.70139

**Published:** 2025-03-26

**Authors:** Diala Haykal

**Affiliations:** ^1^ Centre Laser Palaiseau Private Practice Palaiseau France

**Keywords:** cosmetic dermatology, dermatologybioengineering, skin bioengeeniring, skin structure

Dear Editor,

Cosmetic dermatology is entering a new frontier with the advent of 3D bioprinting, a technology poised to revolutionize the way we approach skin regeneration, aesthetic enhancement, and personalized dermatologic interventions [1]. While initially developed for reconstructive applications, 3D bioprinted skin may soon redefine anti‐aging treatments, scar revision, and even volumetric facial rejuvenation. The ability to print customized skin grafts using a patient's own cells presents an unprecedented opportunity for natural, long‐lasting aesthetic results that go beyond current injectables or energy‐based devices [2]. Unlike synthetic fillers or collagen stimulators, bioengineered skin has the potential to integrate seamlessly with native tissue, not just restoring lost elasticity and improving texture but also offering a truly personalized approach to volume restoration and wrinkle repair. The development of bioinks composed of extracellular matrix components, fibroblasts, and keratinocytes has made it possible to recreate full‐thickness skin layers, closely mimicking natural skin architecture [3]. This breakthrough paves the way for regenerative skin therapies tailored to the patient's unique biology, a major leap from current interventions that primarily focus on temporary improvements. Recent studies have already explored the possibility of bioprinting skin constructs that replicate a patient's biomechanical and pigmentation properties, which could provide highly personalized solutions for ethnic skin tones, hyperpigmentation disorders, and scar remodeling [1, 4]. This could be a game changer in treating post‐inflammatory hyperpigmentation, vitiligo, and burn scars, where uniform repigmentation remains a significant challenge. What if, instead of depigmentation therapies or camouflage techniques, we could bioprint new, pigment‐matched skin? The implications are enormous. Beyond direct aesthetic applications, 3D bioprinting holds promise in testing cosmetic formulations, optimizing post‐procedure recovery, and even hosting patient‐specific microbiomes. Traditional in vitro testing models lack the complexity of real skin, limiting the ability to predict how skincare products, fillers, or energy‐based treatments will interact with human tissue. Bioprinted skin constructs could bridge this gap, serving as functional testing platforms that eliminate the need for animal models and provide more accurate insights into product safety and efficacy [5]. With AI‐driven modeling, the future of personalized skin regeneration becomes even more precise. AI‐assisted tissue engineering could optimize bioprinted scaffold design, ensuring that bioengineered skin is tailored to enhance collagen synthesis, modulate inflammation, and restore dermal architecture [6]. This approach could revolutionize the way we manage atrophic scars, keloids, and aging‐related volume loss, moving us away from temporary solutions toward long‐term skin regeneration. The possibilities are vast, yet surprisingly underexplored in aesthetic research.

Despite its promise, 3D bioprinting raises critical questions about regulatory pathways, accessibility, and ethical considerations. Will these bioengineered skin constructs be classified as medical treatments or luxury enhancements? Could the ability to print customized, youthful skin fuel unrealistic expectations in aesthetic medicine? With designer aesthetics already shaping beauty trends through AI‐driven consultations, how far are we from patients requesting bioprinted modifications tailored to idealized standards rather than functional restoration? These are questions that dermatologists, researchers, and policymakers must address as the technology continues to advance. Another challenge is cost and accessibility. While current dermal fillers and regenerative therapies are widely available, bioprinting remains expensive and largely experimental. Will this technology become a niche service reserved for high‐end cosmetic clinics, or will advancements in biofabrication make it scalable and accessible to a broader population? These considerations must be taken into account as 3D bioprinting transitions from the lab to clinical practice.

With the field of cosmetic dermatology continually evolving, now is the time to start the conversation on how 3D bioprinting could reshape treatment paradigms. The ability to create personalized, regenerative skin therapies presents an exciting challenge, one that could lead to a fundamental shift in aesthetic medicine. As this technology progresses, interdisciplinary collaboration will be crucial in bridging the gap between regenerative medicine, dermatology, and biotechnology. The future of aesthetic medicine may not be about reversing time, but about bioengineering skin to evolve with it. However, further clinical studies and research are essential to fully understand the efficacy, safety, and long‐term potential of bioprinted skin in cosmetic applications.

## Conflicts of Interest

The author declares no conflicts of interest.

## Data Availability

The data that support the findings of this study are available on references' part.
